# Multianalytical Investigation of *Psilocybe cubensis* Mushrooms: Physicochemical Characterization and Biological Evaluation of Psilocybin and Psilocin Compounds

**DOI:** 10.1021/acsomega.5c05606

**Published:** 2025-09-15

**Authors:** Taynah Pereira Galdino, Antônio Braz de Medeiros Bisneto, Marcelo da Silva Pedro, Lucas Cordeiro de Oliveira, Mateus Araújo Luz, Antônio Gilson Barbosa de Lima, Suédina Maria de Lima Silva, Marcus Vinicius Lia Fook

**Affiliations:** † Northeast Biomaterials Evaluation and Development Laboratory, CERTBIO, Academic Unit of Materials Engineering, 154624 Federal University of Campina Grande, 58429-900 Campina Grande, Brazil; ‡ Northeast Biomaterials Evaluation and Development Laboratory, CERTBIO, Postdoc, Federal University of Campina Grande, 58429-900 Campina Grande, Brazil; § Mechanical Engineering Department, Federal University of Campina Grande, Campina Grande 58429-900, Brazil

## Abstract

Mental disorders of the global population have been evaluated statistically by decades. Psilocybin has medicinal properties with potential pharmaceutical applications for treatment of psychological disorders. This component is present naturally in mushrooms of the *Psilocybe* genus and is primarily responsible for their psychoactive properties. It exhibits a low risk of acute toxicity and has shown no evidence of neurotoxicity, carcinogenicity, or mutagenicity in current toxicological assessments. This study aimed to develop an active pharmaceutical ingredient (API) derived from *Psilocybe* mushrooms for application in the management of mental health disorders. The primary objectives include extraction of bioactive compounds, quantitative analysis, and evaluation of their physicochemical and biological properties. Following the extraction procedures, the experiments achieved yields of approximately 20%, demonstrating effective isolation of the target compounds. The presence of alkaloids was confirmed through phytochemical screening and validated by high-performance liquid chromatography (HPLC) analysis. The obtained API exhibited thermal and spectroscopic characteristics consistent with those of edible mushroom extracts, enabling a comprehensive understanding of its physicochemical behavior. Additionally, analytical assays demonstrated high solubility, low toxicity, and compliance with acceptable limits for heavy metal content and microbial load, including aerobic microorganisms, fungi, and yeasts. Quantitative HPLC analysis revealed psilocybin and psilocin contents of 3.26 and 0.34%, respectively, supporting the formulation of drug delivery systems with standardized concentrations of psilocybin. The results indicated that the extracted API is stable, highly pure, and compatible with polymeric matrices for controlled release applications. Furthermore, the assays confirmed high solubility in polar solvents and minimal risk of adverse effects. In conclusion, the study showed that the development of a psilocybin- and psilocin-based API is feasible and represents a significant advancement in the treatment of mental disorders, standing out as a promising solution in the pursuit of innovative therapeutic alternatives.

## Graphical abstract

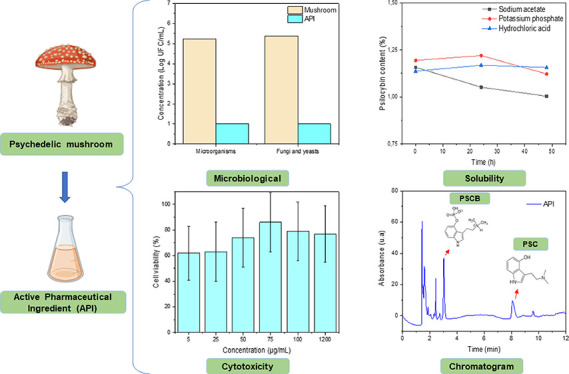

## Introduction

Mental disorders are characterized by clinical conditions that affect cognition, emotional regulation, or behavior, significantly impairing individual functioning and quality of life.^1^ In 2019, approximately 970 million people worldwide were affected by some form of mental disorder, a figure that increased significantly following the COVID-19 pandemic, with anxiety and depression among the most commonly reported conditions. In the vast number of individuals impacted by these pathologies, several therapeutic approaches have been proposed to mitigate or overcome such conditions, including the use of antidepressants, neuromodulation techniques, and psychotherapy. Clinical trials have reported improvements in mental health through psychedelic-assisted psychotherapy, involving substances such as MDMA (3,4-methylenedioxymethamphetamine) for post-traumatic stress disorder (PTSD), LSD (lysergic acid diethylamide) for anxiety, ibogaine for alcoholism, DMT (dimethyltryptamine) for depression, and psilocybin, which has shown broad-spectrum potential due to its favorable safety and efficacy profiles.^2–4^

Recent studies involving *Psilocybe cubensis* have demonstrated that, in animal models, extracts obtained from this fungus enhanced memory and increased levels of brain-derived neurotrophic factor (BDNF) in the hippocampus.^5^ Proteomic analyses revealed the presence of peptides with antimicrobial activity, with potential to inhibit the growth of *Staphylococcus aureus*.^6^ Additionally, recent investigations evaluated the effects of psychedelic compounds present in *P. cubensis* on microglial cells, evidencing modulation of the microglial immune response.^7^ Psychedelic compounds, including psilocybin, also demonstrated efficacy in alleviating symptoms associated with post-COVID-19 syndrome, such as olfactory dysfunction, cognitive deficits, excessive sleepiness, sleep disturbances, and psychological symptoms.^8^

Psilocybin, psychedelic compound naturally found in hallucinogenic mushrooms, acts as a prodrug that is metabolized into psilocin upon oral administration, thereby exerting its psychoactive effects.^9,10^ Clinical studies have explored its therapeutic potential in addressing treatment-resistant depression, generalized anxiety disorder, cancer-related psychological distress, alcohol dependence, and smoking cessation. These effects are attributed primarily to the modulation of brain connectivity and the promotion of neuroplasticity.^11–16^

The therapeutic use of psilocybin and psilocin has driven significant research efforts aimed at elucidating their mechanisms of action, as well as their physicochemical and biological characteristics, with a focus on improving treatment outcomes through optimized formulation strategies.^17–19^ In this context, the development of studies investigating the pharmacological behavior and clinical potential of these compounds is highly relevant for the advancement of medical devices and therapeutic protocols. Thus, under special authorization of the Brazilian Government, the aim of this study was to develop an active pharmaceutical ingredient (API) derived from mushrooms of the *Psilocybe* genus for potential application in the treatment of mental disorders, with a focus on the extraction of bioactive compounds, quantitative analysis, and comprehensive evaluation of their properties.

## Materials and Methods

### Reagents and Standards

The *Psilocybe cubensis* mushrooms used in this study were imported into Brazil (Rosehill Apothecary Ltd., Rose Hill, Jamaica) through an import permit granted by ANVISA (AI 067/2025), specifically for this project, in accordance with the Special Authorization for the study of psilocybin and psilocin (AE No. 30/2024), which includes the control, storage, and handling of these substances. The mushrooms were dried by the supplying company and delivered in hermetically sealed packaging. Psilocybin (1 mg/mL, Lipomed Document QC-CA-411L1) and psilocin (1 mg/mL, Lipomed Document QC-CA-410L1) analytical standards were obtained from LAS do Brasil (Aparecida de Goiânia, Goiás, Brazil). HPLC-grade acetonitrile (ACN) was acquired from Êxodo Científica (Sumaré, São Paulo, Brazil) and SK Chemicals (Seongnam, South Korea), whereas analytical grade formic acid (P.A.) was sourced from Neon (Suzano, São Paulo, Brazil). Ultrapure water (18.2 MΩ·cm, TOC < 10 ppb) was produced via a Milli-Q system (MERCK, model eq 7000, Darmstadt, Germany).

### Experimental Procedure

#### Extraction of Psilocybin and Psilocin

Initially, the dried mushrooms were ground to initiate the extraction process. The dried material was placed in a mortar and submerged in liquid nitrogen. After freezing, the material was pulverized via a pestle until a homogeneous powder was obtained. The resulting powder was then sieved through a 170 mesh (0.088 mm) sieve. The particles retained on the sieve were collected, ground in a mortar, and sieved again until the entire sample reached the desired particle size (<0.088 mm).

The extraction process was carried out via a method adapted from Morita et al.^20^ Initially, the mushroom powder was mixed with an acidified ethanol extraction solution (1–2% acetic acid) at a 1:25 (mL/mg) ratio. The mixture was transferred to amber glass containers and subjected to ultrasonic extraction (Ultronique, Q p.5/40A) for 30 min. Following sonication, the mixture was vacuum-filtered, and the remaining solid was subjected to three additional extraction cycles under the same conditions, totaling four cycles to ensure complete removal of the target compounds. The combined extracts were homogenized and concentrated under reduced pressure via a rotary evaporator (Fisaton, Model 802) at 40 °C until the volume is reduced to less than 100 mL. The resulting concentrate was then transferred to a forced-air oven (Solab, SL-102) at 40 °C to ensure the complete removal of residual solvents. To remove nonpolar compounds, the dry extract was subjected to affinity-based extraction, in which the polarity of the extraction solvent was selected to closely match that of psilocybin.^21^ Hexane was added to the dry extract at a 1:20 (g/mL) ratio and subjected to ultrasonication for 30 min. The hexane supernatant was discarded, and the process was repeated twice with fresh solvent. The remaining solid was then placed in a forced-air oven at 40 °C to eliminate residual hexane, yielding a crude extract.

### Characterization

#### Scanning Electron Microscopy (SEM) and Energy-Dispersive X-ray Spectroscopy (EDS)

Mushroom powder samples were morphologically characterized by scanning electron microscopy (SEM) with TM 1000 HITACHI and PHENOM PROX microscopes. The morphology of the mushroom tissues and pulverized particles was examined at four magnifications (50×, 250×, 1000×, and 2000×), without the need for a gold coating due to the low-voltage operation of the equipment. Chemical characterization was performed through energy-dispersive X-ray spectroscopy (EDS), an essential accessory for identifying the elemental composition of the samples. The analysis of the dried and ground mushroom samples focused on the predominant element and phosphorus contents, with the aim of confirming the presence of psilocybin. Elemental mapping and spectra were obtained at 2000× magnification.

#### X-ray Fluorescence (XRF)

X-ray fluorescence (XRF) analysis was performed via a Malvern Panalytical Epsilon 4 spectrometer capable of detecting elements ranging from carbon (C) to americium (Am) at concentrations ranging from sub ppm levels up to 100% by weight. This technique was applied to mushroom powder samples to determine their elemental composition. The methodology included sample preparation, instrument calibration, X-ray irradiation and detection, spectral analysis, and quality control. The results contributed to meeting quality standards, identifying potential impurities, and optimizing the production process.

#### Simultaneous Thermal Analysis (STA) Coupled with FTIR Spectroscopy

Thermogravimetric analysis (TGA) and differential scanning calorimetry (DSC) were performed to determine the thermal characteristics of the samples. A simultaneous TGA/DSC analyzer (PerkinElmer STA-6000) was used under a nitrogen atmosphere (40 mL/min), heating both the ground mushroom samples and the API from 20 to 900 °C at a rate of 10 °C/min. To investigate the decomposition mechanism, the analyzer was coupled to a Fourier transform infrared (FTIR) spectrometer (PerkinElmer Spectrum 400). The evolved gases were transferred through a heated transfer line (TL 8000) into a gas cell maintained at 270 °C to prevent condensation. The FTIR spectra of the evolved gases were recorded via Spectrum Time Base software in the spectral range of 4000–650 cm^–1^, with a resolution of 4 cm^–1^ and 16 scans per spectrum.

#### Phytochemical Analysis

Phytochemical analyses were performed on both the mushroom sample and its extract, following the methodology widely cited in the literature, as described by Matos.^22^ The presence of saponins was assessed using the foam test, while polysaccharides were detected based on the development of a blue coloration upon addition of Lugol’s iodine solution. Tannins and phenolic compounds were evaluated through colorimetric reactions with FeCl_3_ and gelatin precipitation, enabling the differentiation between hydrolyzable and condensed tannins. Flavonoids were identified using the Shinoda and oxalo-boric reactions, with positive results indicated by the appearance of a red color or greenish-yellow fluorescence under UV light. Steroids and triterpenes were detected through the Liebermann–Burchard reaction, characterized by a color change ranging from evanescent blue to persistent green. The presence of alkaloids was confirmed by precipitation reactions with Bouchardat, Dragendorff, and Mayer reagents. Quinones were identified via the Bornträger reaction, evidenced by a purple coloration in the aqueous phase following chloroform extraction and alkalinization. Finally, coumarins were detected through UV-induced fluorescence after applying NaOH to filter paper moistened with the methanolic extract.

#### Fourier Transform Infrared Spectroscopy (FTIR)

The dried mushroom samples, as well as their macerated forms and extracts, were analyzed via FTIR spectroscopy to identify their components and characteristic vibrational bands. Spectra were acquired in the range of 4000–650 cm^–1^, with 16 scans and a resolution of 4 cm^–1^, using a PerkinElmer Spectrum 400 FT Mid-IR spectrometer.

#### Inductively Coupled Plasma Optical Emission Spectroscopy (ICP-OES)

The assay aimed to determine metal contamination in psilocybin powder and extract samples, classifying it as a trace element analysis. For this purpose, an Optima 8000 inductively coupled plasma optical emission spectrometer (ICP-OES) from PerkinElmer equipped with an axial torch, a nebulizer with a Scott spray chamber, and a charge-coupled device (CCD)-based detection system was used, ensuring the accuracy of the analytical calibration curves. Analyses were conducted in accordance with the United States Pharmacopeia (USP) and ICH Q3D guidelines, adhering to the maximum allowable limits for heavy metals specified in USP ⟨232> and ⟨233⟩.^23,24^ For the analyses, 0.4 g of each sample was weighed in triplicate and subjected to microwave-assisted digestion via the Titan MPS system. The plasma radio frequency power was maintained at 1300 W, and the gas flow rates for the plasma, auxiliary, and nebulizer gases were set at 8.0, 0.2, and 0.7 L/min, respectively.

#### Microbiological Profile Analysis

Microbiological analysis was performed to detect the presence of aerobic microorganisms, molds, and yeasts. The method employed was the pour plate technique, in which a 1:10 (sample/sterile water) suspension is prepared and serial dilutions ranging from 1/10 to 1/10,000 are conducted. For the enumeration of aerobic microorganisms, 1000 μL of each dilution was transferred into Petri dishes, followed by the addition of 15 mL of casein soy agar (CSA) melted and maintained at 50 °C. The plates were incubated in an inverted position at 35 °C for 3 days. For molds and yeasts, Sabouraud dextrose agar was used, and the plates were incubated at 22 °C for five to 7 days. After the incubation period, the number of colony-forming units (CFUs) was counted and recorded.

#### Cytotoxicity

The assay was based on ISO 10993-5:2009 and was used to evaluate the in vitro cytotoxicity of medical devices. The L929 fibroblast line (ATCC NCTC clone 929), obtained from the Rio de Janeiro Cell Bank, Brazil, was used in the test. The colorimetric cytotoxicity assay was performed via the MTT assay (3-(4,5-dimethylthiazol-2-yl)-2,5-diphenyltetrazolium bromide), which assesses mitochondrial dehydrogenase activity to determine cell viability. Optical density readings were taken using a Victor X3 spectrophotometer (PerkinElmer) at 570 nm, with a reference filter at 650 nm. Cell viability was calculated as a percentage, and a modified z score test was applied to identify outliers. Different concentrations of API were tested (5, 25, 50, 75, and 100 μg/mL), along with a 1.2 mg/mL concentration, as recommended by the standard.

#### High-Performance Liquid Chromatography Coupled with Diode Array Detection (HPLC-DAD)

The identification and quantification of psilocybin and psilocin were performed via a mobile phase composed of acetonitrile (ACN, HPLC grade) and ultrapure water (H_2_O), both of which were acidified. ACN was filtered through a nylon membrane, and water was passed through an *N*-acetylcellulose membrane. Both solvents were acidified with 0.3% formic acid. For quantification, 1 mg of API was dissolved in 950 μL of acidified ultrapure water and 50 μL of acidified acetonitrile, followed by manual agitation. The sample was filtered through a 0.22 μm syringe filter and transferred to an HPLC vial. For samples derived from API, the same preparation procedure was followed. Analyses were carried out via a PerkinElmer LC-FLEXAR high-performance liquid chromatography (HPLC) system coupled with a diode-array detector (DAD) and a SOGEVAC vacuum pump (model SV40BI). Identification and quantification were performed via a calibration curve employing a C18 column (150 mm length, 4.6 mm internal diameter, 5 μm particle size). The column oven was maintained at 30 °C, the injection volume was 15 μL, and the mobile phase flow rate was set at 0.8 mL/min.

The identification of psilocybin and psilocin in the API was performed via a validated method, in which the quantification of the substances was determined via eq 1 for psilocybin (*C*_PSCB_) and eq 2 for psilocin (*C*_PSC_). *C*_PSCB_ and *C*_PSC_ are given in mg/L.

$$C_{\mathrm{PSCB}}=\frac{A_{\mathrm{PSCB}}+106,505}{18,959}$$ 1

$$C_{\mathrm{PSC}}=\frac{A_{\mathrm{PSC}}+106,505}{18,959}$$ 2

where *A*_PSCB_ and *A*_PSC_ represent the peak areas obtained for psilocybin and psilocin, respectively.

#### Solubility

The solubility assay was performed via the equilibrium method, following the Dissolution Guide for Generic, New, and Similar Drugs.^25^ The objective was to evaluate the solubility of the API under various physiological pH conditions (ranging from 1.0 to 6.8) to understand how this chemical parameter influence the release of the API.^26^

The procedure involved the addition of an excess amount of API to obtain saturated solutions, which were maintained under controlled agitation at 37 ± 1 °C for 24 to 48 h until thermodynamic equilibrium was reached. The media selected for this evaluation were 0.1 mol/L hydrochloric acid (pH 1.2), 0.1 mol/L sodium acetate buffer (pH 4.5), and 5 mmol/L potassium phosphate buffer (pH 6.8). In each flask, 40 mL of the respective solution was added, followed by incremental addition of the API extract until the solvent reached saturation, as indicated by undissolved material settling at the bottom of the container. Approximately 5 mg of psilocybin was used, corresponding to an estimated 0.43 mg of psilocin within that API mass.

The analysis was carried out via the shake-flask method with a shaker incubator (IKA KS 4000i), maintaining constant agitation at 200 rpm and 37 °C for 24 h. During the incubation period, 1 mL samples were collected at 24 and 48 h, and the volume was replaced with 1 mL of blank solvent. After collection, the samples were filtered through 0.22 μm polyvinylidene fluoride (PVDF) membranes and quantified via high-performance liquid chromatography (HPLC). On the basis of the concentrations obtained, the dose/solubility (D/S) ratio was calculated from the amount of API used and the measured concentration, as described in eq 3.^27^

$$\frac{D}{S}=\frac{\mathrm{Mass}\;\mathrm{used}\;(\mathrm{mg})}{\mathrm{Measured}\;\mathrm{concentration}\;(\mathrm{mg}/\mathrm{mL})}$$ 3

## Results and Discussion

### Scanning Electron Microscopy (SEM) Combined with Energy-Dispersive X-ray Spectroscopy (EDS)

Scanning electron microscopy (SEM) was performed to analyze the morphology of the *Psilocybe* mushrooms. The mushroom was sectioned into three parts: the upper region of the cap (fraction 1), the gills located on the underside of the cap (fraction 2), and the stipe or stem (fraction 3), which were analyzed at magnifications of 250× and 1000× (Figure 1). From the micrographs, it is possible to observe that fraction 1 has a rough surface profile, likely due to mushroom dehydration. At a magnification of 1000×, microfibers and light-colored particles can be observed, which may be associated with the mineral components present in the mushroom. In the gill region (fraction 2), at 250× magnification, a smooth surface with scattered dots is observed. When magnified to 1000×, these structures are identified as mushroom spores, some of which appear ruptured, whereas others remain intact, displaying a morphology similar to that of red blood cells. Analysis of the stipe revealed a fibrous appearance, with fibers oriented in the direction of mushroom growth—characteristic of the stems of plants and fungal materials.

**Figure 1:**
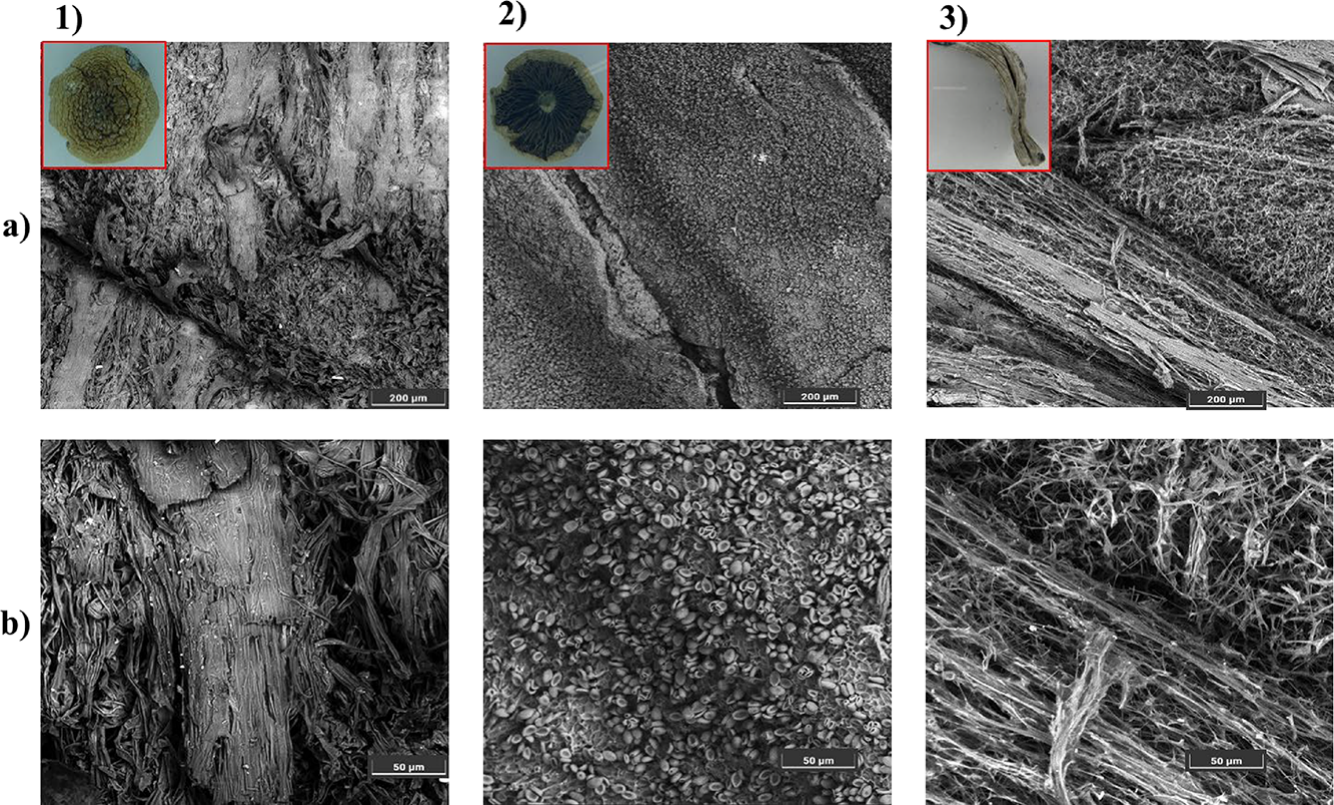
Micrographs of the samples from (1) the outer cap surface, (2) the inner cap (gill region), and (3) the stipe at magnifications of (a) 250× and (b) 1000×.

The particle morphology of the mushroom powder was analyzed at 250× magnification, along with elemental composition analysis via energy-dispersive X-ray spectroscopy (EDS), in order to estimate the approximate elemental content, with a particular emphasis on phosphorus, an element present in the chemical structure of psilocybin, as shown in Figure 2. The micrographs revealed particles of varying sizes, with the powder sample exhibiting predominantly rough and fibrous surfaces, corresponding to the cap and stipe regions, respectively. The EDS spectrum displays ionization energy and counts, where higher counts indicate greater presence of a given element. On the basis of the SEM–EDS micrograph, the predominant chemical elements were found to be spatially distributed throughout the analyzed section, with carbon, potassium, oxygen, and phosphorus highlighted in red, purple, green, and blue, respectively—elements commonly associated with fungal matrices. Additionally, trace amounts of chlorine, magnesium, iron, aluminum, and silicon were also detected. Although the elemental composition of hallucinogenic mushrooms has not been widely reported in the literature, these mushrooms can be analyzed similarly to edible mushrooms, allowing for the mapping and identification of major chemical constituents.

**Figure 2:**
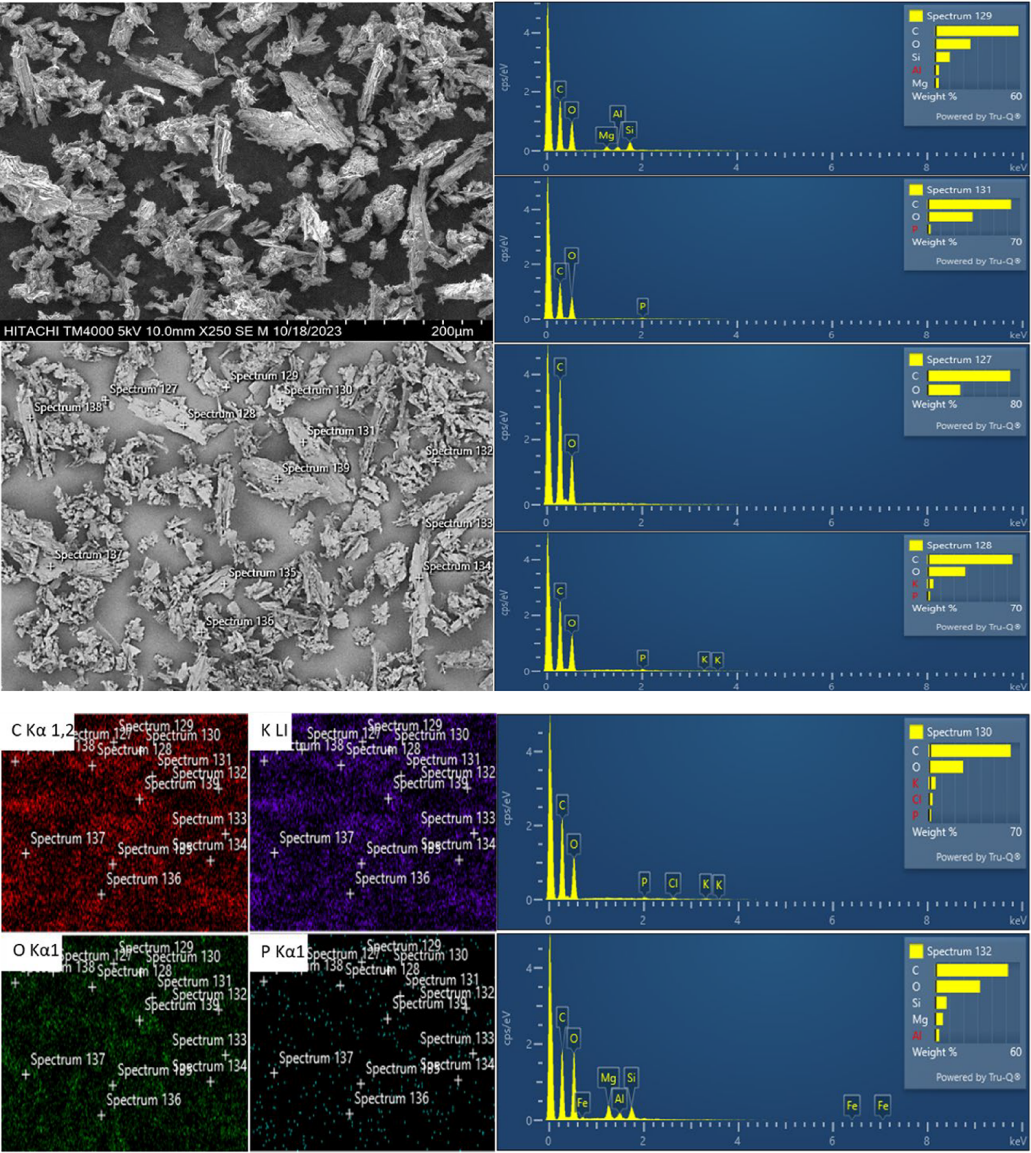
Micrograph and EDS analysis of the macerated mushroom at 250× magnification. On the left, elemental distribution maps for carbon, oxygen, potassium, and phosphorus. On the right, the elemental composition at selected points within the image shows the corresponding EDS spectra with peak intensities for each detected element.

Studies on edible mushrooms have reported the presence of elements such as calcium, iron, magnesium, phosphorus, potassium, sodium, zinc, copper, manganese, and selenium.^28,29^ However, in the present study, energy-dispersive X-ray spectroscopy (EDS) analysis did not detect calcium, sodium, zinc, copper, manganese, or selenium. Additionally, previously listed elements, including chlorine, aluminum, and silicon, were identified. The presence of chlorine may be attributed to phenolic compounds, such as pentachlorophenol.^30^ Muñoz, Corona, Wrobel, Soto and Wrobel^31^ investigated the subcellular distributions of aluminum, bismuth, cadmium, chromium, copper, iron, manganese, nickel, and lead in cultivated mushrooms, and reported the presence of aluminum localized in the cell walls of certain species. Silicon, on the other hand, has not been widely documented in the literature, although it is a common component of sand and may appear as a contaminant introduced during cultivation, harvesting, storage, or processing. Although EDS elemental analysis provides useful insights into the spatial distribution of key elements, complementary analytical techniques are necessary for more accurate characterization.

### X-ray Fluorescence (XRF)

Macronutrients such as carbon (C), hydrogen (H), sodium (Na), potassium (K), chlorine (Cl), and calcium (Ca), among others, are typically present at concentrations in the percentage range or in milligrams per gram (mg/g). Micronutrients, or trace elements, such as iron (Fe), zinc (Zn), selenium (Se), and cobalt (Co), among others, are found at significantly lower concentrations, typically in the microgram per gram (μg/g) to nanogram per gram (ng/g) range.^32^ Figure 3 shows the X-ray fluorescence (XRF) spectrum obtained from the atomic ionization of the elements detected in the powdered sample of *Psilocybe* genus mushrooms. Table 1 presents the elemental analysis and the corresponding percentage compositions of the identified elements.

**Figure 3:**
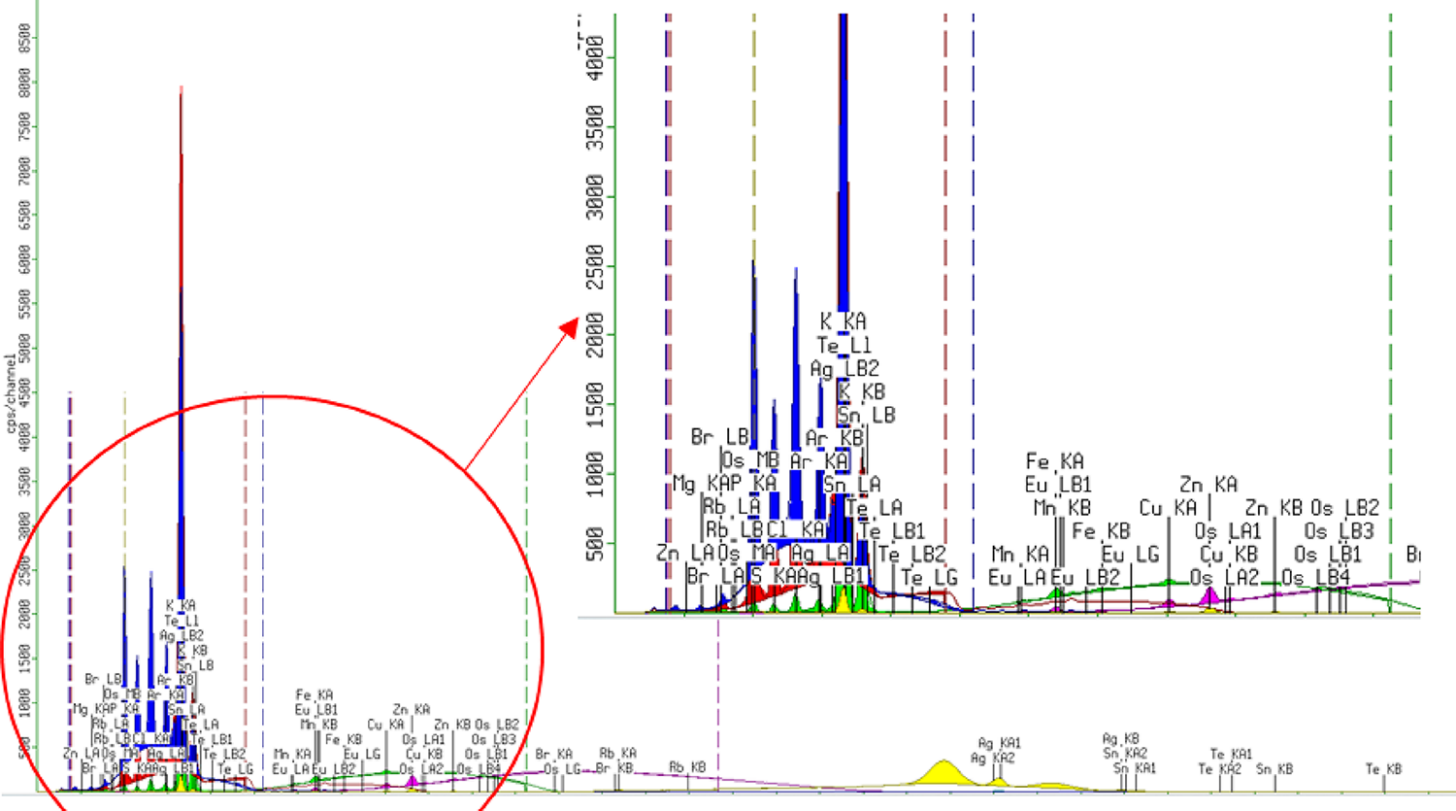
XRF spectra of macerated *Psilocybe* mushroom samples.

**Table 1:** Elemental and Compositional Analysis of Macerated Psilocybe Mushrooms Performed via X-ray Fluorescence (XRF)

element	(%)	compound	(%)
Mg	1.177	MgO	1.677
P	13.278	P_2_O_5_	24.333
S	4.086	SO_3_	7.711
Cl	5.233	Cl	3.856
K	75.310	K_2_O	61.699
Mn	0.074	MnO	0.058
Fe	0.310	Fe_2_O_3_	0.270
Cu	0.134	CuO	0.101
Zn	0.311	ZnO	0.234
Br	0.007	Br	0.004
Rb	0.028	Rb_2_O	0.019
Sn	0.025	SnO_2_	0.019
Te	0.025	TeO_2_	0.019
Os	0.001	OsO_4_	0.001

According to the obtained data, the *Psilocybe* mushroom contains a variety of identified chemical elements, including magnesium, phosphorus, sulfur, chlorine, potassium, manganese, iron, copper, zinc, bromine, rubidium, tin, tellurium, and osmium. Mineral composition analysis revealed the predominant presence of potassium oxide (61.7%) and phosphates (P_2_O_5_ at 24.33%), also confirmed by EDS analysis. These were followed by sulfur trioxide (7.7%), chlorine (3.9%), magnesium oxide (1.7%), and other elements present at concentrations below 1%. The identified elements do not exhibit inherent toxicity; however, manganese (Mn), iron (Fe), copper (Cu), and zinc (Zn) may be potentially toxic if they are consumed in excessive amounts. The remaining elements (Mg, P, S, Cl, K, Br, Rb, Sn, Te, and Os) are essential to human physiology when present at appropriate concentrations and are generally not considered toxic at normal dietary levels, indicating that the sample is nontoxic.^33^ The detection of P_2_O_5_ suggests the presence of psilocybin in the mushroom, highlighting its potential for the extraction of psilocybin with yields suitable for the development of active pharmaceutical ingredients (APIs) based on this compound.

An investigation of wild medicinal mushrooms of the species *Ganoderma lucidum* by Popa et al.^34^ revealed the presence of the chemical elements Si, P, Fe, Ag, Ca, K, Mn, Sr, S, and Ni in most of the collected samples. However, a wide range of additional elements was also detected, including Ag, Al, As, At, Br, Ca, Cl, Co, Cu, Eu, Fe, Fr, Ga, Gd, Ge, Ir, K, Lu, Mn, Mo, Mg, Ni, Os, P, Pb, Po, Pt, Rb, Re, S, Se, Si, Sr, Ti, Tm, U, V, W, Y, and Zn, highlighting the compositional variability among mushrooms. The most significant mineral content observed in this species included iron and/or potassium. In a similar study on wild mushrooms, Turhan et al.^35^ quantified the levels of K, Fe, Cu, Mn, Zn, Pb, Cd, Ni, Sn, Br, Sr, Ti, Rb, As, Th, and U across 11 species of wild mushrooms. Among all the analyzed samples, not all contained elements such as zinc, cadmium, nickel, selenium, bromine, rubidium, arsenic, thorium, and uranium. Potassium was consistently the most abundant element, which is in agreement with the findings of the present study. However, iron was reported as the second most abundant element in Turhan’s study, which contrasts with the results obtained for *Psilocybe*, where the iron content was notably lower in comparison.

### Simultaneous Thermal Analysis (STA-FTIR)

The powdered *Psilocybe* mushroom and the obtained active pharmaceutical ingredient (API) were analyzed via simultaneous thermal analysis (STA), and Fourier transform infrared (FTIR) spectra of the evolved gases, which were collected during sample heating. On the basis of thermogravimetric analysis (Figure 4), which is supported by its derivative curve, five distinct mass loss events can be identified. The first mass loss, observed in the TGA curve (black line), occurred between 30–117 °C, with a maximum at 73 °C, as indicated by the DTG peak (red line). The second event occurred between 117–162 °C, with a peak at approximately 140 °C, which corresponds to the elimination of adsorbed water bound to the matrix and physically retained moisture, despite the samples being dried. The third mass loss takes place between 162–217 °C, with a maximum at 200 °C, that is associated with the volatilization of low-molecular-weight compounds. The fourth event, between 220–375 °C, with a peak at 307 °C and a shoulder at 338 °C, corresponds to the pyrolysis of organic components, which is related primarily to polysaccharides and microbial cell wall constituents, especially cellulose and hemicellulose, as well as the release of more thermally stable volatiles. Finally, the fifth mass loss begins at approximately 375 °C and extends to approximately 500 °C, with a noticeable shoulder, and is attributed to the combustion of macromolecular cellulose derivatives and the presence of thermally resistant compounds such as lignin.

**Figure 4:**
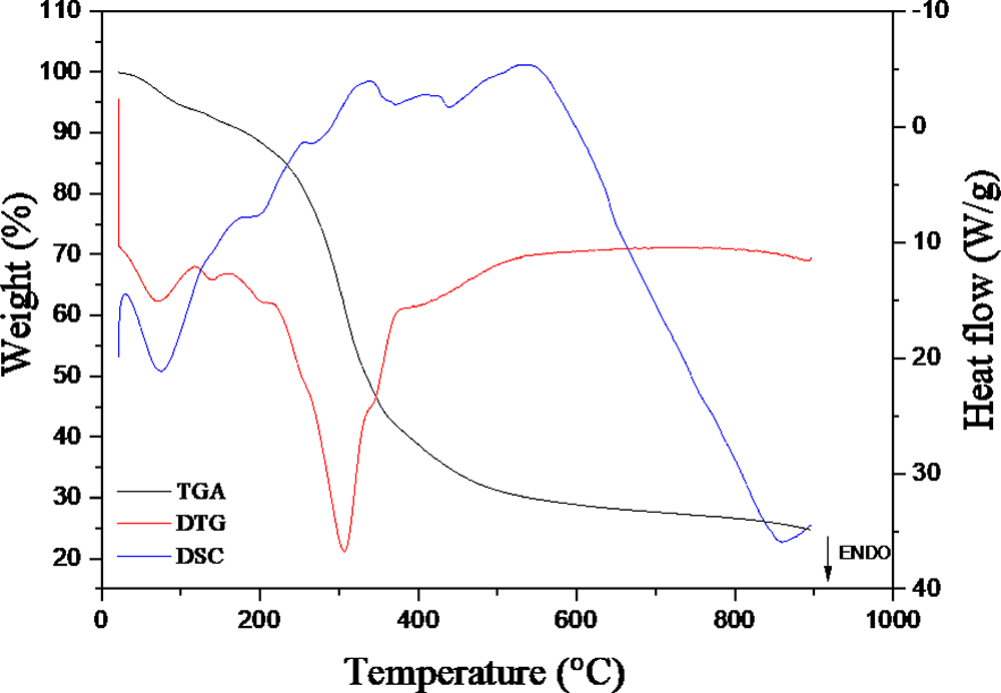
TGA-DTG-DSC curves of the whole mushroom powder sample.

As observed by Sharma,^36^ mass loss during the thermal degradation of mushroom constituents can be attributed to the successive combustion of components such as cellulose, xylan, chitin, microbial biomass, and phenolic compounds. The author anticipated thermal responses between 200–420 and 420–580 °C, corresponding to primary and secondary peaks, respectively, as seen in the DTG curve (Figure 4). The results revealed water loss at approximately 90–110 °C, a major weight loss between 200–420 °C associated with hemicellulose and microbial cell walls, a minor peak near 420 °C attributed to structural hemicellulose, and a secondary activity peak between 420–580 °C linked to the shoulders or decomposition of more stable compounds, such as lignin. These findings are consistent with those observed for the *Psilocybe* mushroom in the present study. However, while Sharma identified only one water-related mass loss event, two distinct losses were observed here, possibly influenced by different heating rates during analysis. Ming et al.^37^ evaluated the thermal properties of *Lentinus edodes* mushrooms and identified three mass loss stages via thermogravimetric analysis: the first stage occurred between 35–200 °C, the second stage occurred between 200–350 °C, and the third stage occurred between 350–590 °C. The first was related to the loss of adsorbed water and the decomposition of thermolabile compounds, the second was related to the oxidative degradation of organic matter and volatile components, and the third was related to the combustion of cellulose macromolecules. The mass loss events reported by these authors are in agreement with those identified in the present study.

On the basis of DSC analysis Figure 4, five thermal events were identified: the first two peaks are endothermic, followed by three exothermic events. The first endothermic event begins at approximately 30 °C and extends to 120 °C, with a peak temperature at 76 °C and an enthalpy change (Δ*H*) of 93.04 J/g, corresponding to the release of adsorbed water. The second endothermic transition occurs between 172–225 °C, peaks at 203 °C, with an enthalpy of 11.09 J/g, and is associated with the volatilization of low-molecular-weight compounds. The subsequent exothermic events occurred in the ranges of 236–266, 290–372, and 437–646 °C, with peak temperatures at 252, 335, and 551 °C, and enthalpy changes of Δ*H* = −3.44, Δ*H* = −35.14, and Δ*H* = −267.10 J/g, respectively. These exothermic peaks correspond to the thermal decomposition and degradation of organic constituents. The events between 250–400 °C are attributed mainly to the decomposition of cellulose and hemicellulose, whereas the degradation of lignin occurs at temperatures above 400 °C, characterized by continuous mass loss.^38^

The particle processing conditions of *Tremella fuciformis* mushroom powder were investigated by Tsai et al.^39^ These researchers reported that smaller particle sizes provided greater thermal stability, as evidenced by DSC and TGA analyses. The endothermic events identified by the authors involved varying onset temperatures, reflecting the influence of particle size on the hydration behavior and thermal stability of the samples. However, only one thermal event of each type (endothermic or exothermic) was reported. In comparison, the peak temperature of the endothermic event observed in the present study was lower than the range,^39^ with a smaller enthalpy requirement, suggesting a reduced water content in the sample. Moreover, the volatilization event was not detected by those authors, possibly because of the co-occurrence of moisture loss and volatilization at lower temperatures, resulting in a single thermal event. With respect to the exothermic event, the peak temperature of 252 °C found in this study was within the range observed by Tsai et al.,^39^ but the enthalpy was significantly lower. Additionally, three distinct exothermic events were identified here, likely due to the compositional variability of the organic constituents in the material.

On the basis of the STA analysis, infrared spectra were collected at the characteristic temperatures corresponding to the mass loss peaks observed in the TGA/DTG curves and the thermal events identified via DSC in order to elucidate the structures of the volatilized substances, as shown in Figure 5. The analysis of gases released during thermal events, conducted via thermogravimetric analysis coupled with FTIR spectroscopy, enabled the identification of volatile compounds emitted throughout the thermal degradation process of both whole mushrooms and their extracts. This integrated approach, which combines TGA/DTG/DSC data with FTIR spectral information, provides a comprehensive understanding of the thermal and chemical transformations occurring within these materials.

**Figure 5:**
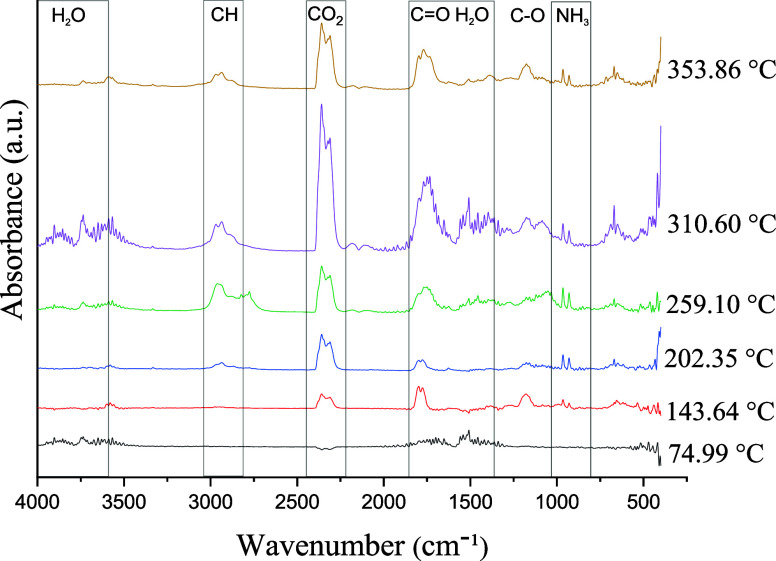
FTIR spectra of gases evolved during STA analysis of the mushroom powder collected at approximately 75, 143, 202, 310, and 353 °C.

In the mushroom powder, the initial mass loss observed between 30–117 °C, with a maximum at 75 °C, was associated with the release of adsorbed and chemically bound water within the material matrix. This event was corroborated by the presence of an infrared absorption band at approximately 1640 cm^–1^, corresponding to the bending vibration of water molecules. Alongside the second mass loss, which occurs between 117–162 °C and peaks near 140 °C, the continued presence of water, both physically adsorbed and chemically bound, was confirmed—although with a reduced intensity compared with that of the previous stage. In this interval, new absorption bands emerged within the 1750–1850 cm^–1^ and 2250–2400 cm^–1^ regions, indicating the onset of thermal decomposition of volatile organic compounds or residual carbonyl-containing groups, such as aldehydes and ketones. The FTIR spectrum associated with the third mass loss event, observed at 202 °C in the analysis of the whole mushroom, revealed more defined bands in the 2800–3000 cm^–1^ range, corresponding to the stretching vibrations of C–H bonds in methyl and methylene groups, suggesting the release of volatile compounds such as alcohols and low-molecular-weight hydrocarbons. Concurrently, the bands in the 1700–1750 cm^–1^ region became more pronounced, suggesting further elimination of aldehydes and ketones. As the temperature increased (approximately 260 °C), characteristic polysaccharide bands began to dominate the spectra, particularly within the 1000–1200 cm^–1^ range, corresponding to C–O stretching vibrations linked to sugar decomposition. Additional bands in the 1500–1600 cm^–1^ region indicated the formation of aromatic compounds, likely arising from the thermal breakdown of cell wall components such as cellulose and hemicellulose. In higher temperature ranges (310.60 and 353.88 °C), the spectra exhibited a sharp increase in absorption bands within the 2300–2400 cm^–1^ region, attributed to CO_2_ evolution—a major combustion product of thermally stable organic matter such as lignin. Persistent bands in the 1500–1600 cm^–1^ region remained evident and were associated with the degradation of complex aromatic structures. This thermal behavior supports the progressive combustion of organic residues, highlighting the relatively high thermal stability of lignin.^40,41^

Like the thermogravimetric analysis (TGA) and differential scanning calorimetry (DSC) results obtained for the mushroom powder, the analysis of the mushroom extract (Figure 6) enabled the identification of several thermal events related to its chemical composition and thermal behavior. The TGA curve revealed three major stages of mass loss for the extract. The first mass loss occurred within the temperature range of 90–230 °C and was characterized by a prominent DTG peak at approximately 110 °C, accompanied by a set of lower-intensity peaks throughout this interval. This event is attributed to the release of residual water, including both physically adsorbed and chemically bound water, as well as the volatilization of residual compounds—possibly related to the presence of secondary metabolites or solvent residues from the extraction process—which were less evident in the analysis of the raw mushroom material. While this thermal behavior resembled that observed in the raw sample, the transition in the extract appeared more concentrated, indicating that the extraction process reduced the heterogeneity of the material matrix. Additionally, previous studies reported the volatilization of phenolic compounds and terpenes within this temperature range, further supporting the interpretation of this thermal event.^42–44^

**Figure 6:**
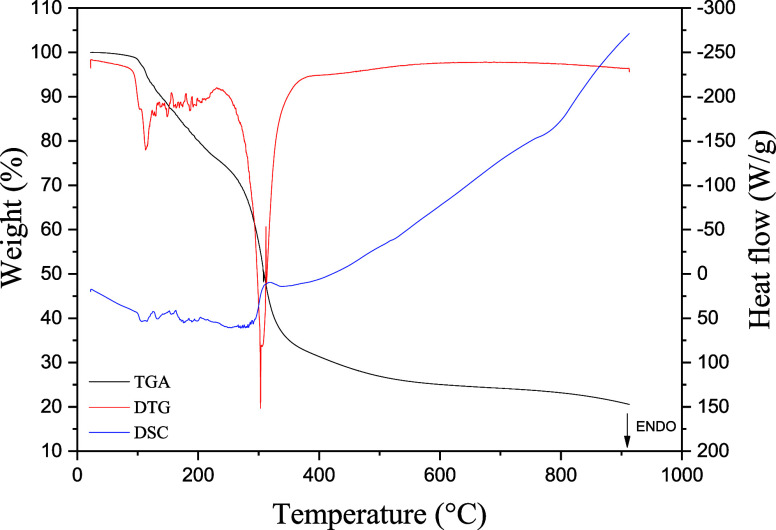
TGA-DTG-DSC curves of the extract sample (API).

The second mass loss occurred between 240–400 °C, with a prominent DTG peak at 300 °C and a secondary peak at 312 °C. This thermal event is associated with the degradation of organic compounds, particularly polysaccharides extracted from mushroom cell walls. Previous studies have reported that constituents such as cellulose and hemicellulose undergo significant thermal decomposition within this temperature range.^45^ Furthermore, the DSC curve (in blue) displays an exothermic peak in this interval, supporting the occurrence of structural component degradation. The third stage of mass loss takes place between 400–600 °C, with a shoulder at approximately 450–500 °C, indicative of the combustion of thermally stable macromolecules such as lignin and other recalcitrant phenolic compounds. The presence of these compounds is also evident in the analysis of the raw mushroom sample but appears to be more concentrated in the extract, suggesting that the extraction process enriched the content of these thermostable constituents. Finally, the residual mass observed above 600 °C corresponds to the mineral fraction or ash content, representing the inorganic compounds present in the extract.

DSC analysis complements this interpretation by revealing both endothermic and exothermic transitions. The endothermic peak near 100 °C is attributed to water evaporation, whereas the exothermic events observed at approximately 350 and 450 °C are associated with the degradation and combustion of organic compounds. The absence of more pronounced endothermic peaks above150 °C suggests that the compounds present in the extract exhibit high thermal stability or were already released during earlier stages. To gain a better understanding of the substances volatilized during the thermal process, the FTIR spectra of the compounds released at the peak temperatures of the thermal events were analyzed, as shown in Figure 7.

**Figure 7:**
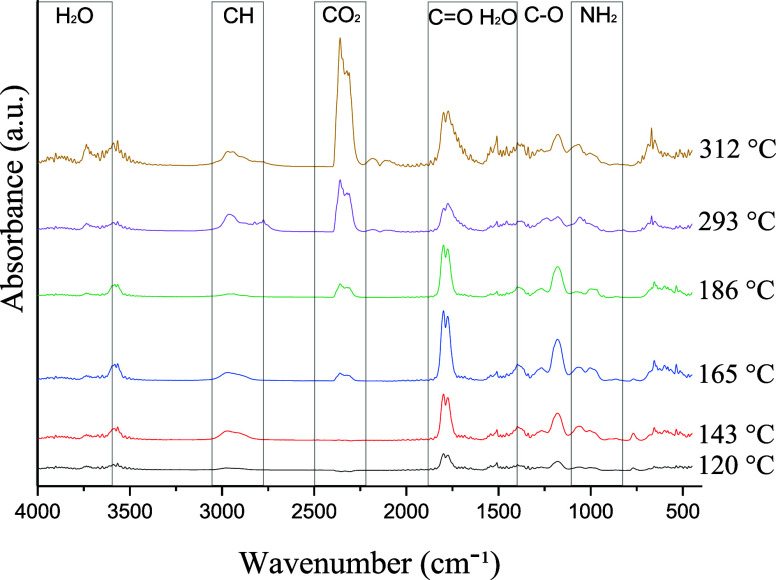
FTIR spectra of the gases released during the STA analysis of the API collected at different temperatures.

In the initial temperature range of 120 °C, a band between 1720–1840 cm^–1^ is observed, which is typical for C=O stretching vibrations. This suggests the release of volatile aldehydes, ketones, or carboxylic acids. In the region between 1130–1231 cm^–1^, corresponding to C–O bonds frequently observed in alcohols, ethers, or ester-containing compounds, the spectra indicate the initial decomposition of polysaccharides or other extract components containing oxygen bonded to carbon chains. This behavior differs slightly from that of the whole mushroom, where the dehydration of adsorbed water dominates the early thermal events. As the temperature increased to 143 °C, the bands at approximately 3000, 1700–1750, and 1000–1200 cm^–1^ remained highly intense, confirming the emission of organic compounds derived from the initial decomposition of molecules present in the extract. At 165 and 186 °C, there was an intensification of the bands in the 2800–3000 cm^–1^ region, confirming the release of aliphatic hydrocarbons. More defined bands in the 1700–1750 cm^–1^ range indicate the liberation of carbonyl compounds, whereas the absorptions between 1000–1200 cm^–1^ reflect C–O vibrations characteristic of alcohols and esters. Upon reaching 293 °C, the band in the 2300–2400 cm^–1^ region becomes significantly intensified, characterizing the predominant release of CO_2_—a typical product of carbohydrate thermal decomposition. In this interval, the absorption in the 1500–1600 cm^–1^ range indicates the presence of aromatic compounds, suggesting the onset of lignin degradation as well as the breakdown of other thermostable components. The residual bands in the 1000–1200 cm^–1^ region still reflect the decomposition of the remaining polysaccharides. Finally, in the temperature range of 312 °C, the band in the 2300–2400 cm^–1^ region dominated, reflecting the massive release of CO_2_ during the combustion of thermostable macromolecules.^40,41^

Compared with the raw mushroom, the extract exhibited a more clearly defined profile of thermal events, reflecting the concentration of specific compounds present during the extraction process. In whole mushrooms, the release of adsorbed water occurs at slightly lower temperatures, indicating lower moisture retention than in the extract. Furthermore, the presence of polysaccharides and lignin is more pronounced in the whole mushroom, as demonstrated by the higher intensity of bands related to C–O vibrations and CO_2_ emissions at intermediate temperatures, revealing the characteristics of a more heterogeneous matrix with a broader range of mass loss temperatures. In contrast, in the extract, the increased intensity of bands in the 1700–1750 cm^–1^ region reflects the concentration of volatile and bioactive compounds—possibly enriched during the extraction process—which characterize it as a more homogeneous material, thereby facilitating the identification of volatile compounds, polysaccharides, and lignin.

### Phytochemical Analysis

The qualitative phytochemical analyses (Table 2) of the mushroom in powder form revealed the following: saponins were detected by the formation of foam; polysaccharides were evidenced by a blue color shift; tannins and phenolic compounds were indicated by the appearance of a blue coloration; flavonoids were identified exclusively through the oxalo-boric reaction, with fluorescence observed under ultraviolet light; and alkaloids yielded positive results in all tests, as demonstrated by a color change in the Bouchardat and Dragendorff reactions, along with the formation of a white precipitate in the Mayer test. Further, steroids and terpenoids were identified on the basis of the observed color changes. No traces of other metabolites, such as quinones or coumarins, were detected. In the fungal extract (API), only the steroids, terpenoids, and saponins were not extracted; that is, the *Psilocybe* mushroom extract contains tannins, phenolic compounds, flavonoids, alkaloids, and polysaccharides—with the indication of polysaccharides showing only a slight color change, suggesting a low concentration of these compounds.

**Table 2:** Qualitative Results of the Phytochemical Tests on the Psilocybe Mushroom Powder and API Obtained

test	mushroom powder	API
saponins	positive	negative
polysaccharides	positive	positive
tannins and phenolic compounds	positive	positive
flavonoids	positive	positive
alkaloids	positive	positive
steroids and terpenoids	positive	negative
quinones	negative	negative
coumarins	negative	negative

The presence of alkaloids, tannins, phenolic compounds, polysaccharides, and flavonoids in the extract confirms the occurrence of bioactive principles that may exhibit medicinal properties. Psilocybin and psilocin are alkaloids; therefore, the extraction method used in this research is assumed to be efficient in isolating the target substances. These results agree with the findings of Dhanasekaran et al.*,* ^46^ who conducted a taxonomic identification of bioactive compounds in *Psilocybe* mushrooms. They analyzed the crude extract and observed the presence of saponins, tannins, flavonoids, and alkaloids, differing only with respect to the detection of saponins. Moreover, the authors reported that steroids and terpenoids were not detected, which corroborates the results obtained for the *Psilocybe* mushroom extract. Margret, Mareeswari, Kumar and Jerley^47^ investigated the presence of phytochemicals in extracts of *Agaricus bisporus* and *Pleurotus ostreatus*, identifying saponins, tannins, phenolic compounds, terpenes, steroids, flavonoids, glycosides, alkaloids, and amino acids. The only divergence observed was in the identification of steroids, while the presence of the other compounds was consistent with the substances identified in the present study.

### Fourier Transform Infrared Spectroscopy (FTIR)

The FTIR analysis of the mushroom, as shown in Figure 8, reveals important information about its molecular composition, particularly regarding the presence of proteins and polysaccharides. The spectrum shows a prominent absorption band between 3400 and 3000 cm^–1^, which corresponds to the vibrational contributions of −NH bonds and, primarily, to the symmetric and asymmetric stretching vibrations of −OH groups. These differences are attributed to water molecules and carbohydrate structures. In addition to stretching, water also exhibits an infrared-active bending vibration related to the opening and closing of the H–O–H bond angle, which appears at approximately 1640 cm^–1^. This band may overlap with other molecular vibrations, such as the C=O stretching of amide groups. The region between 2900 and 2850 cm^–1^ is associated with the stretching vibrations of methyl and methylene groups (CH_2_/CH_3_), likely originating from polysaccharides and lipids. This is further supported by the absorption near 1150 cm^–1^, attributed to glycosidic linkages, as well as the region between 1080–1000 cm^–1^.

**Figure 8:**
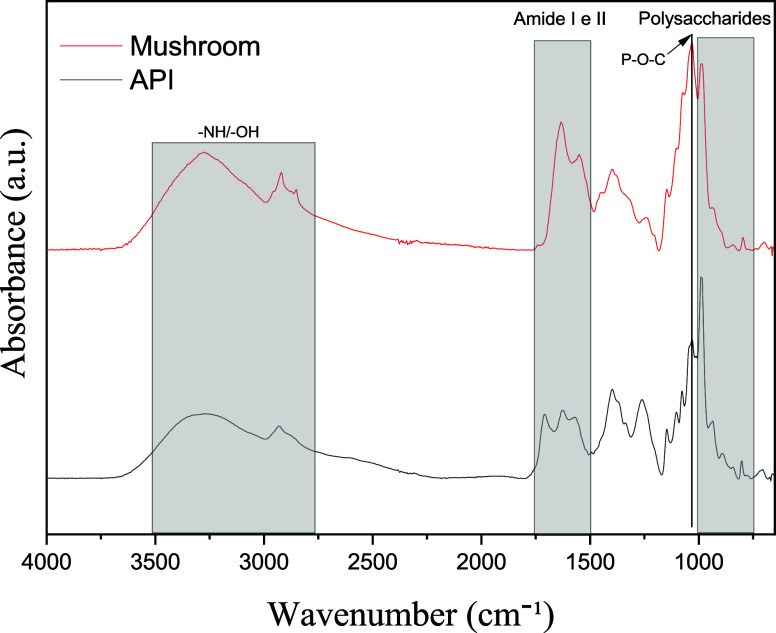
Comparative FTIR spectrograms of the mushroom powder and API.

On the basis of the spectral analysis, a comparative assessment can be made with data reported in the literature to better understand the spectroscopic behavior of the mushroom, as shown in Table 3, and to establish a comparison with the obtained extract. A comparison of the current data with literature data revealed that the absorption band at 1635 cm^–1^ is associated with the amide I vibration, corresponding to the C=O stretching of proteins, which is indicative of the presence of peptide bonds. The band at 1543 cm^–1^ corresponds to the amide II vibration, which is also related to protein components. Typically, this frequency appears at a lower wavenumber than the C=O stretching of carboxylic acids or esters, suggesting a low contribution of lipid content in this fungus.

**Table 3:** Comparison between Vibrational Bands and Their Corresponding Wavenumbers: Literature versus Experimental Observations

	wavenumber (cm^–1^)	
vibration bands	Esteves et al.^48^	observed in the experiments
stretching −OH/-NH	3271	3272
asymmetric stretching −CH	2918–2925	2918/2850
amide I	1633–1636	1635
amide II	1539–1543	1543
bending of −CH in −CH_2_/-CH_3_	1455	1455
bending modes of–CH e −COH	1400–1413/1368–1393/1312	1400/1360/1308
C–C stretching/–CO stretching from acids and esters	1243–1253	1232
C–O–C stretching vibrations	1147–1153	1151
–CO and C–C stretching/P–O–C stretching	1077	1072
–CH deformation (polysaccharides)	1019–1030/929/890	1033/942/891

The presence of bands at approximately 1400 cm^–1^ is attributed to out-of-phase CH bending and COH bending vibrations, whereas the band at 1368 cm^–1^ is associated with in-phase C–H bending. In the region of 1232 cm^–1^, the band can be linked to C–C stretching of the pyranose ring and to stretching vibrations of carboxylic acids or ester −CO groups, suggesting the presence of cyclic structures and potential steric interactions. At lower frequencies, approximately 1153 cm^–1^, the C–O–C stretching vibration of the glycosidic linkage appears, which is characteristic of polysaccharide structures. A broad and intense band at 1077 cm^–1^ is assigned to the −CO stretching of alcohol groups, which are predominantly found in polysaccharides. This band may also encompass contributions from C–C stretching and possibly −POC group vibrations in the presence of trace amounts of psilocybin or lipids. Additionally, a weak band observed at 890 cm^–1^ may be related to β-linked polysaccharides, particularly due to C–H deformation or β-glycosidic linkages.^48^

These results indicate that *P. cubensis* has a complex matrix rich in proteins and polysaccharides, with minimal evidence of lipids or fatty acids, reflecting its biochemical composition and both structural and bioactive functionalities. Compared with the obtained ATR-FTIR spectrum, a similar pattern can be observed, with variations in absorption bands within the 1750–1200 cm^–1^ range, corresponding to the amide I and amide II bands of proteins, as well as carboxylic acid and ester vibrations. These findings suggest a limited or absent extraction of these compound classes. Furthermore, the definition of the P–O–C absorption band suggests a higher concentration of compounds containing phosphate ester linkages.

### Inductively Coupled Plasma Optical Emission Spectrometry (ICP-OES)

The concentrations of arsenic, cadmium, mercury, and lead were quantified, with reference values provided according to the lowest permissible limits established by the International Council for Harmonization (ICH)^49,50^ and the Brazilian Pharmacopoeia (BP),^51^ as shown in Table 4. On the basis of the obtained results, all four analyzed elements are below the limits specified by both regulatory standards used as a reference for this study, demonstrating the material’s purity in terms of heavy metal content for both the raw material (mushroom powder) and the obtained extract (API). Furthermore, the data revealed a reduction in metal content after the extraction process, with cadmium levels reduced by 100% and lead levels reduced by approximately 50%. This suggests that the extraction process does not fully transfer metallic elements to the final product, which is favorable for the intended biomedical applications of the material.

**Table 4:** Quantification of Heavy Metals in Comparison with the Limits Established by the International Council for Harmonization (ICH) and the Brazilian Pharmacopoeia (BP)

element	mushroom powder	API	lower ICH limit	BP
As (μg/g)			<0.2	<5.0
Cd (μg/g)	0.044 ± 0.006	0.000 ± 0.000	<0.2	<1.0
Hg (μg/g)	0.000 ± 0.020	0.000 ± 0.000	<0.1	<0.1
Pb (μg/g)	0.092 ± 0.025	0.041 ± 0.025	<0.5	<5.0

A bibliometric analysis of European publications between 2001 and 2016 conducted by Świsłowski et al.^52^ regarding the concentrations of various elements in mushrooms indicated that cadmium was the most frequently discussed element. The highest cadmium concentration reported in *Paxillus involutus* was 3964 mg/kg. The highest concentration of lead was observed in *Amanita citrina*, at 895 mg/kg, followed by *Macrolepiota procera*, at 171 mg/kg. Although these concentrations exceed those reported in the present study, the mushrooms analyzed by the authors were collected from regions with probable environmental, which may account for the variation in phytochemical content. *Moura* ^53^ performed heavy metal determination in 24 samples across three mushroom genera (*Pleurotus*, *Lentinus*, and *Agaricus*) via triplicate measurements and calculated average concentrations of multiple elements. Among the four elements previously mentioned, only arsenic was quantified, with concentrations ranging from 0.020 to 0.206 μg/g in *Lentinus*, 0.094 to 0.402 μg/g in *Agaricus*, and 0.027 to 0.080 μg/g in *Pleurotus*. Compared with those in the *Psilocybe* mushroom powder analyzed in this study, the arsenic levels observed were lower than those.^53^ Falandysz et al.^54^ carried out an elemental analysis of 38 elements in wild mushrooms from Poland. The authors reported relatively low concentrations of lead (0.54–1.3 μg/g), cadmium (0.56–12 μg/g), and mercury (0.19–5.5 μg/g), with arsenic not being quantified. In comparison, the cadmium content in *Psilocybe* mushrooms analyzed in the present study was below the minimum level reported by Falandysz and colleagues, lead concentrations were within the same range, and mercury was not detected in our sample.

### Microbiological Profile Analysis

The microbiological evaluation of the mushroom powder and the obtained IFA extract was based on the enumeration of aerobic microorganisms, yeasts, and molds, following the pour plate method. The results are presented in Table 5. Microbiological analysis revealed that microbial loads tend to be lower in the material after the extraction process, with a significant reduction in CFUs. The aerobic microorganism count decreased from 5.23 log CFU/mL to values below 1 log CFU/mL, whereas the fungal count decreased from 5.38 log CFU/mL to less than 1 log CFU/mL, indicating that the extracted material contained a markedly lower total microbial burden. Considering that mushroom powder is a raw natural product, the CFU levels observed exceeded the limits established by the Brazilian Pharmacopoeia. However, this material can be classified as untreated or non-preprocessed. In contrast, the API showed microbial counts well below all the pharmacopoeial limits, regardless of the classification criteria, highlighting its potential for biomedical applications under appropriate microbial quality control standards.

**Table 5:** Quantitative Results of Microorganism, Fungal, and Yeast Counts in Mushroom Powder and API

	unit	mushroom powder	API
total aerobic microorganisms	UFC/mL	17 × 10^4^	<10
	log UFC/mL	5.23	<1
fungi and yeasts	UFC/mL	24 × 10^4^	<10
	log UFC/mL	5.38	<1

In a study conducted by Venturini et al.,^55^ 402 samples from 22 species of fresh and wild mushrooms were evaluated. The microbial counts ranged from 4.4 to 9.4 log CFU/g, with no significant differences observed among the species. The average values of the yeast population were between 3.2 and 3.7 log CFU/g. Compared with the samples analyzed in the present study, the total microbial count fell within the range reported by the authors, although notably lower than the median values observed. In contrast, the counts for yeasts and molds were relatively high. Kim et al.^56^ assessed the microbial load in commercial shiitake mushrooms, identifying aerobic microorganism levels ranging from 3.3 to 7.5 log CFU/g and yeast and mold counts between 2.2 and 6.0 log CFU/g. When compared, the values obtained for the materials analyzed in the present study fall within the ranges reported by these authors.

### Cytotoxicity

The cell growth viability of the API was assessed through an in vitro fibroblast (L929) cell assay via the MTT test, which serves as a reliable marker for evaluating the cytotoxicity of bioactive compounds. The cells were cultured and exposed to the API at concentrations of 5, 25, 50, 75, 100, and 1200 μg/mL. The assay was conducted in 12-well plates, and the average standard deviation was calculated, as shown in Figure 9. Polyphenolic compounds, along with polysaccharides, proteins, and their complexes, may exert cytotoxic effects in mushroom extracts, depending on their concentration, molecular interactions, and extraction conditions.^57,58^ Among these groups, the obtained API demonstrated the presence of unquantified polyphenols and proteins, which may contribute to reduced cell viability. Therefore, it is necessary to evaluate the potential cytotoxicity these constituents may exert on the material.

**Figure 9:**
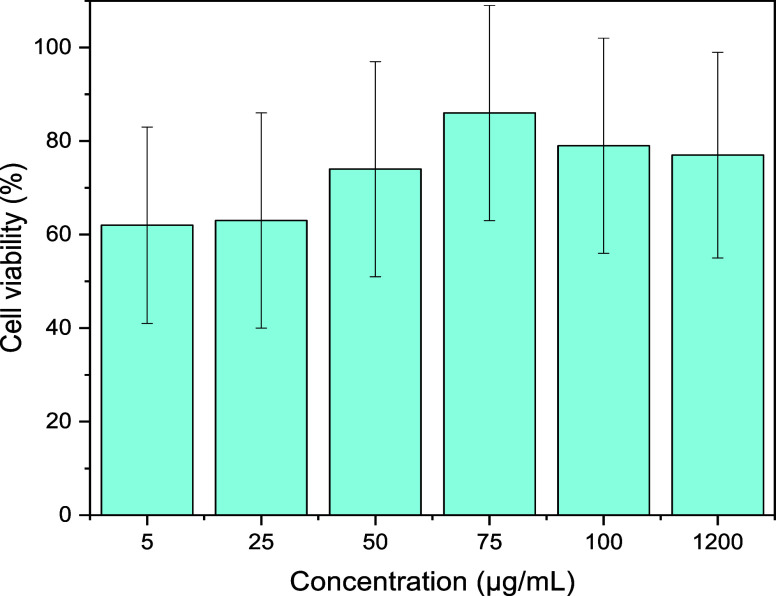
Cell viability assessment at different concentrations of the API (5, 25, 50, 75, 100, and 1200 μg/mL).

On the basis of the cell viability results, lower concentrations of the API (<50 μg/mL) resulted in viability levels below 70%, whereas concentrations above this threshold maintained cell viability above 74%. The highest concentration tested (1.2 mg/mL or 1200 μg/mL) resulted in a cell viability profile comparable to that of the other concentrations, indicating the absence of cytotoxic effects even at high doses. These findings suggest a low toxicity threshold for the studied extract, reinforcing its potential for pharmaceutical applications.

A study conducted by Taofiq et al.^59^ evaluated the effects of ethanolic extracts of Ganoderma lucidum and Pleurotus ostreatus on keratinocyte and fibroblast viability at various concentrations. The viability of fibroblasts was maintained at 60% at 100 μg/mL for G. lucidum and at 90% for P. ostreatus. Similar to the trend observed with Ganoderma, the API in the present study resulted in cell viability above 60% at concentrations up to 100 μg/mL, but unlike G. lucidum, it maintained these levels at considerably higher concentrations (up to 1200 μg/mL). Nkadimeng et al.^60^ assessed the safety and therapeutic potential of psychedelic mushrooms such as Psilocybe cubensis and Panaeolus cyanescens from the genera Psilocybe and Panaeolus under conditions of pathological hypertrophy. These results indicated that aqueous extracts of P. cyanescens and P. cubensis did not worsen endothelin-1-induced pathological hypertrophy and provided protection against TNF-α-induced injury and cell death at the concentrations tested. With respect to cell viability, the extracts maintained levels above 80% at concentrations of 10 and 25 μg/mL. In the present study, viability was lower at these concentrations but remained close to 80% at concentrations above 50 μg/mL, reaching its peak at 75 μg/mL and slightly decreasing at higher concentrations.

### High-Performance Liquid Chromatography Coupled with Diode Array Detection (HPLC-DAD)

High-performance liquid chromatography (HPLC) was employed to identify and quantify the presence of psilocybin and psilocin in the obtained API. Qualitative identification of the compounds was carried out and confirmed through the injection of analytical standards of psilocybin (PSCB) and psilocin (PSC), along with the API sample, with the absorbance monitored at 266 nm, as shown in Figure 10. Additionally, UV–Vis spectra were retrieved for the peaks corresponding to PSCB and PSC in the analytical standards, as well as for the peak associated with the compounds detected in the API sample, providing detailed information regarding their UV–Vis absorption profiles, as illustrated in Figure 11. This approach allowed for comprehensive analysis of the chromatographic behavior of the injected samples, thereby confirming the presence of psilocybin and psilocin in the API.^61^

**Figure 10:**
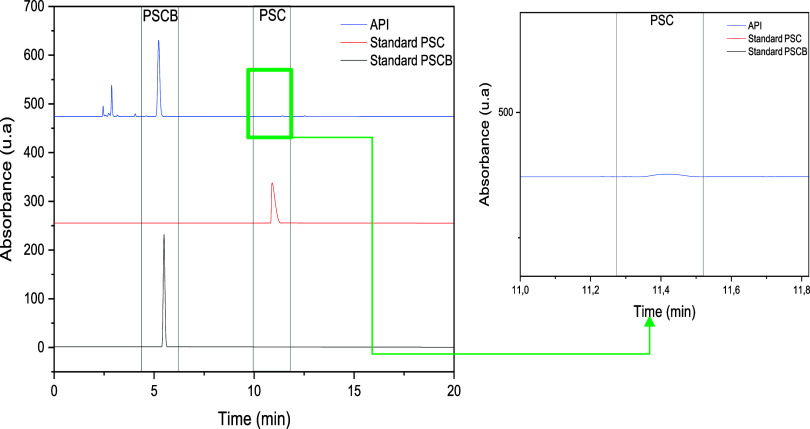
Representative HPLC chromatograms of standard psilocybin (PSCB), psilocin (PSC), and the obtained active pharmaceutical ingredient (API).

**Figure 11:**
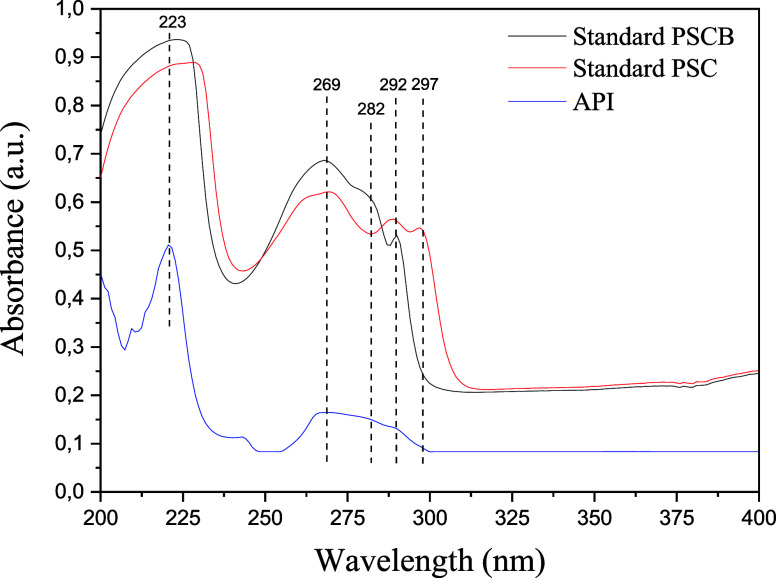
Comparison of UV–Vis spectra for standard compounds (PSCB and PSC) and the obtained API.

The retention of the PSCB and PSC standards is consistent with their respective molecular polarity properties, which results in distinct retention times due to their specific interactions with the chromatographic column. Moreover, the chromatogram of the extract shows peaks corresponding to those of the PSCB and PSC standards, indicating the presence of these compounds in the API. The PSCB peaks in the extract appear with moderate intensity, whereas the PSC peak appears as a baseline deviation, suggesting that this compound is present in very low amounts. Additional minor peaks, which do not match those of the standards, may indicate the presence of other compounds in the extract, such as secondary metabolites or polysaccharides. With respect to retention time, the compounds were eluted within the expected range, with only minor variations that do not compromise the reliability of the results. To confirm the presence of these substances in the API, the UV–Vis absorption profiles revealed spectral similarity between the standards and the extract, providing greater confidence in the results presented in Figure 11.

As shown in Figure 11, psilocybin exhibited a main absorption band at 223 nm, attributed to the π–π electronic transition in the aromatic systems of its structure, specifically within the indole rings derived from tryptophan. Psilocin also displayed a band at 223 nm, indicating the preservation of the aromatic moiety but with a relatively high absorption intensity. This difference may be explained by the absence of the phosphoryl group in psilocin, which enhances electronic conjugation and increases interaction with UV radiation. Additionally, psilocin exhibited secondary absorption bands between 260 and 300 nm, with peaks at 269, 282, 292, and 297 nm, characteristic of aromatic systems with extended conjugation and the presence of hydroxyl and amine functional groups. On the other hand, the API extract displayed a less intense band at 223 nm, likely due to the low concentrations of psilocybin and psilocin or interference from other matrix constituents, such as proteins, carbohydrates, and secondary metabolites. This interference may obscure the characteristic absorption bands of the target compounds, thereby increasing the complexity of the spectral interpretation of the extract.

HPLC-UV analysis of psilocin and psilocin revealed that the main peaks of these compounds occur between 220–230 nm, whereas secondary bands from 260–300 nm are more prominent in psilocin. However, significant interferences were observed in the crude extracts due to the presence of other matrix components.^62^ According to The Merck Index,^63^ psilocybin has maximum absorption at 220 nm with secondary absorption at 267 and 290 nm, whereas psilocin has maximum absorption at 222 nm, along with additional peaks at 260, 263, 283, and 293 nm. These values are consistent with the results obtained in the present analysis, in which the standards exhibited an absorption band between 260 and 280 nm. Additionally, a shoulder at 282 nm was observed in the psilocybin standard, a feature not previously reported in The Merck Index,^63^ and a band at approximately 292 nm was detected in both standards, corresponding to absorptions at 290 nm for psilocybin and 293 nm for psilocin.

These findings are also supported by the literature, such as the study conducted by Christiansen and Rasmussen,^64^ which reported UV–Vis absorption peaks at 219, 266, and 288 nm, as well as a shoulder at 280 nm in the spectra obtained from psilocybin standards. For psilocin standards, the authors identified absorption peaks at 221, 266, 282, and 292 nm. These values corroborate the experimental data obtained in this study, reinforcing the reliability of the results and their consistency with previously published findings. Chromatographic and UV–Vis spectral data (Figures 10 and 11) confirmed the presence of psilocybin and psilocin in the API (Active Pharmaceutical Ingredient), enabling the quantification of these compounds in the extract. Quantification of Table 6 was performed via a validated method in accordance with the guidelines established by RDC No. 166/2017, which allows for the determination of compound percentages based on the area under the curve (AUC) of the specific peaks, as described by eqs 1 and 2.

**Table 6:** Quantification of Psilocybin and Psilocin in the API derived from Psilocybe Mushroom

	area	concentration (mg/L)	injected mass (mg)	percentage (%)
PSCB	512799.59	32.66	1.0	3.26
PSC	11911.49	3.46	1.0	0.34

The API obtained from the extraction of mushrooms belonging to the *Psilocybe* genus presented an average psilocybin content of approximately 3.26% ± 0.05%, with psilocin levels of approximately 0.34% ± 0.05%. These values correspond to an average of 6.53 mg of psilocybin and 0.69 mg of psilocin per gram of dried mushroom, exceeding the concentrations commonly reported in the literature. According to Stijve and Kuyper,^65^ the psilocybin content in *Psilocybe* mushrooms may range from 0.1 to 1.3% dry weight, whereas psilocin levels are typically lower, varying between 0.01 and 0.35%. Gartz, Allen, and Merlin^66^ reported psilocybin concentrations of up to 2% in cultivated species, indicating that cultivation parameters, as well as harvesting and processing conditions, may significantly influence the concentrations of these bioactive alkaloids. Laussmann and Meier-Giebing^67^ reported 1.151 ± 0.228 mg of psilocybin and 0.126 ± 0.066 mg of psilocin per 100 mg of dry weight. The elevated psilocybin levels observed in the API may be attributed to multiple factors, including species-specific metabolic profiles, the cultivation environment, the developmental stage at harvest, drying protocols, and extraction methodologies.

### Solubility

Information regarding the solubility of the API in dissolution media within the physiological pH range contributes to the risk assessment associated with biowaiver decisions, using the Biopharmaceutics Classification System (BCS) as a reference criterion.^51^ The samples subjected to the solubility assay were quantified via high-performance liquid chromatography (HPLC) through the collection of 1 mL aliquots at time zero (representing the solubility equilibrium condition) and subsequently at 24 and 48 h. Psilocybin and psilocin concentrations were determined in sodium acetate buffer (SAB), sodium phosphate buffer (SPB) and hydrochloric acid (HCl), as shown in Figure 12.

**Figure 12:**
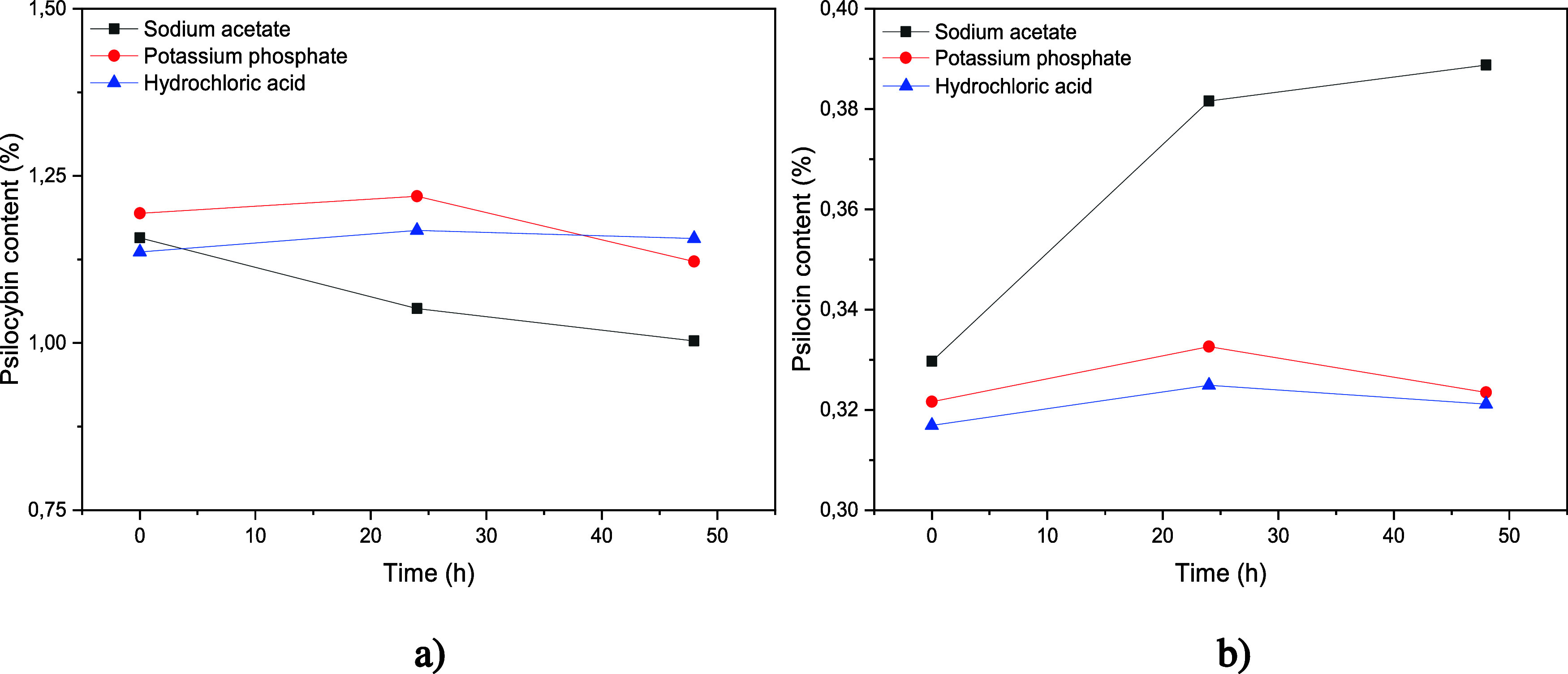
Concentration percentages of psilocybin (a) and psilocin (b) at 0.24 and 48 h in sodium acetate buffer (black line), sodium phosphate buffer (red line), and hydrochloric acid solution (blue line).

Chromatographic analysis demonstrated the stability of psilocybin in all three tested media at 0.24 and 48 h intervals. The chromatograms indicated that the concentration of the active compound remained essentially unchanged over time, with only minor fluctuations, thereby indicating its resistance to degradation under the experimental conditions of the solubility assay. Based on eqs 1 and 2, the concentration of psilocybin in each tested medium (expressed in mg/mL) was used to calculate the dose/solubility ratio (D/S) according to eq 3. The D/S data enabled the determination of sink conditions, and the results of the solubility analysis are presented in Table 7.

**Table 7:** Solubility of Psilocybin and Psilocin Determined via the Shake-Flask Method

dissolution medium	psilocybin concentration(mg/mL)	ratio D/S (mL)	psilocin concentration(mg/mL)	sink condition (mL)
HCl 0.1 M	0.107 ± 0.008	46.71	0.037 ± 0.003	140.143
SAB pH 4.5	0.118 ± 0.005	42.42	0.032 ± 0.008	127.262
SPB pH 6.8	0.115 ± 0.002	43.35	0.032 ± 0.000	130.058

The results revealed no significant differences in solubility across the tested media for either psilocybin or psilocin. Psilocybin exhibited the highest solubility at pH 4.5 (0.118 mg/mL), with slightly lower values at pH 6.8 (0.115 mg/mL) and pH 1.2 (0.107 mg/mL). Psilocin, which is more apolar than psilocybin, showed lower solubility in all media, with only minor variations among them. Psilocybin demonstrated greater solubility under mildly acidic conditions, suggesting that this pH range may be more suitable for conventional oral formulations, such as capsules and tablets. When compared directly, psilocin is less soluble because of its greater lipophilicity.

The D/S ratios were found to be below the 250 mL threshold, which classifies psilocybin as a high-solubility drug across all tested pH media, according to the Biopharmaceutics Classification System (BCS). Therefore, it can be categorized as a Class I compound with high solubility and high permeability. Additionally, psilocybin recovery data revealed 85.6% in HCl, 94.32% in SAB, and 92.24% in SPB. Although the operational conditions were not optimal, the results suggest that psilocin also has D/S values below 250 mL, indicating that it may likewise be classified as a high-solubility compound.

The *sink* condition, which assesses whether the volume of solvent available in the body is sufficient to maintain drug dissolution, was recalculated for a 25 mg dose. Typically, dissolution medium volumes of 500, 900, and 1000 mL are used. However, higher volumes may be required for poorly soluble compounds.^68^ Considering a volume of 900 mL, the solubility results under *sink* conditions were determined, as presented in Table 8.

**Table 8:** Solubility and *Sink* Condition Results

dissolution medium	psilocybin concentration (mg/mL)	*Q* ^a^ (mg)	VN^b^ (mL)	VCs^c^ (mL)	sink condition (ratio 900 mL/VN)
HCl 0,1 M	0.107 ± 0.008	96.3	233.65	700.93	3.85
SAB pH 4.5	0.118 ± 0.005	106.2	211.86	635.59	4.25
SPB pH 6.8	0.115 ± 0.002	103.5	217.39	652.17	4.14

a *Q* = amount of API dissolved in 900 mL of dissolution medium (psilocybin solubility × 900 mL).

b VN = volume required to dissolve 25 mg of psilocybin.

c VCs = volume required to meet *sink* conditions (3 × VN).

All *sink* conditions were greater than 3, indicating sufficient capacity in all cases to ensure complete drug dissolution within typical gastrointestinal fluid volumes. These results demonstrate that, even at its maximum dose (25 mg), psilocybin meets the criteria for high solubility as defined by the Biopharmaceutical Classification System (BCS). However, the variability in *sink* conditions across different pH values highlights the importance of tailoring formulations to the specific solubility and pH-dependent characteristics of drugs. For the medium containing hydrochloric acid, although the *sink* condition remained above 3, it represented a moderate *sink* environment—sufficient to support continuous psilocybin dissolution under acidic conditions, such as those found in the stomach. Nevertheless, the acidic medium had the lowest *sink* value among the tested media, which suggests that the acidic medium has a relatively limited capacity to maintain excess drug in solution.

The acetate buffer exhibited the highest *sink* condition among the three media analyzed, with a value of 4.25. This suggests a slightly greater capacity to maintain dissolution equilibrium than the other media do. However, a pH of 4.5 is less representative of physiological conditions within the gastrointestinal tract, as most drug absorption occurs at pH values closer to neutral, such as those found in the small intestine. The phosphate buffer, which simulates the environment of the small intestine, presented a *sink* condition of 4.14—compared with that of the acetate buffer. This result is particularly relevant, as the small intestine is the primary site of psilocybin absorption and its conversion to psilocin, the active metabolite. The moderately high solubility observed at this pH suggests that phosphate buffer would be an ideal medium for dissolution testing, as it more accurately reflects physiological conditions.

## Conclusions

In this article, an in-depth investigation into the extraction and quantification of psilocybin and psilocin from *Psilocybe cubensis* mushrooms was conducted. The aim is to highlight the therapeutic potential of these compounds for application in the treatment of mental health disorders. The results obtained in this study demonstrated the feasibility of developing an API of psilocybin and psilocin with high therapeutic potential for the treatment of mental disorders. The extraction methodology using fungal matrices was effective, yielding approximately 20% by mass, indicating the presence of alkaloids, tannins, phenolic compounds, polysaccharides, and flavonoids. High-performance liquid chromatography (HPLC) played a critical role in quantifying the target compounds in the API, with the psilocybin content measured at 3.26% and the psilocin content measured at 0.34%. Thermal analysis revealed well-defined thermal events, including initial release of volatile oxygenated compounds, followed by polysaccharide decomposition, emission of aliphatic hydrocarbons, and degradation of thermostable macromolecules. Compared with the raw mushroom matrix, the extract contained higher concentrations of bioactive and volatile compounds, reflecting a more homogeneous thermal profile characteristic of the extraction process. Microbiological analyses (aerobic microorganisms, yeasts, and molds), toxicological assessments in fibroblasts, and heavy metal screening revealed low toxicity and the absence of significant adverse effects, reinforcing the safety of the compound for clinical use. Furthermore, solubility profiling demonstrated the high affinity of the API for polar solvents, increasing its bioavailability. The use of the whole mushroom extract, characterized by a multifaceted composition, has proven to be fundamental for the beneficial effects observed to date. Taken together, these findings support the feasibility of developing safe and effective therapeutic formulations based on this API for the treatment of mental health disorders.
